# Abnormal activation patterns in MT+ during visual motion perception in major depressive disorder

**DOI:** 10.3389/fpsyt.2024.1433239

**Published:** 2024-08-26

**Authors:** Dong-Yu Liu, Xi-Wen Hu, Jin-Fang Han, Zhong-Lin Tan, Xue Mei Song

**Affiliations:** ^1^ Affiliated Mental Health Center & Hangzhou Seventh People’s Hospital, Interdisciplinary Institute of Neuroscience and Technology, School of Medicine, Zhejiang University, Hangzhou, China; ^2^ Key Laboratory of Biomedical Engineering of Ministry of Education, Qiushi Academy for Advanced Studies, College of Biomedical Engineering and Instrument Science, Zhejiang University, Hangzhou, China

**Keywords:** major depressive disorder, percent signal change, task-state fMRI, middle temporal visual cortex, visual motion perception

## Abstract

**Objective:**

Previous studies have found that patients with Major Depressive Disorder (MDD) exhibit impaired visual motion perception capabilities, and multi-level abnormalities in the human middle temporal complex (MT+), a key brain area for processing visual motion information. However, the brain activity pattern of MDD patients during the perception of visual motion information is currently unclear. In order to study the effect of depression on the activity and functional connectivity (FC) of MT+ during the perception of visual motion information, we conducted a study combining task-state fMRI and psychophysical paradigm to compare MDD patients and healthy control (HC).

**Methods:**

Duration threshold was examined through a visual motion perception psychophysical experiment. In addition, a classic block-design grating motion task was utilized for fMRI scanning of 24 MDD patients and 25 HC. The grating moved randomly in one of eight directions. We examined the neural activation under visual stimulation conditions compared to the baseline and FC.

**Results:**

Compared to HC group, MDD patients exhibited increased duration threshold. During the task, MDD patients showed decreased beta value and percent signal change in left and right MT+. In the sample comprising MDD and HC, there was a significant negative correlation between beta value in right MT+ and duration threshold. And in MDD group, activation in MT+ were significantly correlated with retardation score. Notably, no such differences in activation were observed in primary visual cortex (V1). Furthermore, when left MT+ served as the seed region, compared to the HC, MDD group showed increased FC with right calcarine fissure and surrounding cortex and decreased FC with left precuneus.

**Conclusion:**

Overall, the findings of this study highlight that the visual motion perception function impairment in MDD patients relates to abnormal activation patterns in MT+, and task-related activity are significantly connected to the retardation symptoms of the disease. This not only provides insights into the potential neurobiological mechanisms behind visual motion perception disorder in MDD patients from the aspect of task-related brain activity, but also supports the importance of MT+ as a candidate biomarker region for MDD.

## 1 Introduction

Major depressive disorder (MDD) is a severe mental disorder characterized by a high incidence rate, recurrent episodes, and a high suicide rate (1), but its etiology and pathological mechanisms are unclear. A large number of potential biomarkers dominate the progression of the disease, causing patients to exhibit a variety of symptoms, including slowness in behavior, also known as retardation (2, 3). A well-known visual paradigm can reflect the processing of visual motion information in the human higher-order occipital middle temporal complex (MT+) (4, 5). MT+ is a crucial region in the brain for processing motion perception, essential for understanding motion direction and speed (4–6). Our previous study found that MDD patients exhibited abnormal visual motion perception (7). The abnormal performance of MDD patients in this domain may indicate a reduction in their ability to process dynamic visual information, a capability that is vital for responding to moving objects in daily life. However, there is a lack of related task-state studies targeting this function in MDD patients, and the underlying neural mechanisms are currently unclear. Our goal is to study the neural activation pattern in MDD patients during the perception of motion stimuli and its relationship with behavioral manifestations of visual perceptual abnormalities.

Task-state functional magnetic resonance (task-state fMRI) allows for the observation of brain activity in various brain regions during the execution of related visual motion tasks. A classic fMRI task can elicit neural activation in MT+ through visual stimuli of moving gratings (5, 8). Research on suppression and facilitation neural mechanisms revealed that, compared to small grating stimuli, large grating stimuli evoke significant suppression in fMRI response in both early visual cortex (EVC) and MT+ (5). In another study, they found that higher baseline levels of glutamate in MT+ enhance motion perception through elevated neural responses in this region (8). These findings suggest that this fMRI task serves as an available tool for exploring neural activation patterns related to MT+ and primary visual cortex (V1).

Currently, there are several studies that have explored the application of task-state fMRI in visual tasks for depression, including the emotion processing task (9–13), emotional facial recognition task (14–17), Go-NoGo task (18), memory task (19, 20), Stroop task (21–23), learning task (24), visual related task (25) and visual attention task (26, 27). However, fMRI studies on depressed patients performing visual motion processing tasks are very limited, potentially requiring further scientific exploration.

Our previous research found that MDD patients exhibited reduced Glu (glutamate) and GABA in left MT+ (7) and increased Amplitude of Low Frequency Fluctuation (ALFF) as well as abnormal functional connectivity (FC) in resting state (28). However, it remains unclear whether there would be changes in the activation pattern of MT+ during visual motion perception task in MDD patients. In this study, we utilized psychophysical experiment and ultra-high field 7 T MRI to explore the brain function of the visual cortex in MDD patients during a visual grating motion perception task, based on the classic task-state fMRI (5, 8). We hypothesize that, compared to the healthy control (HC), MDD patients would exhibit impaired performance in psychophysical experiment and reduced neural activity during fMRI task. Considering the relationship between resting-state ALFF in MT+ and retardation score identified in previous research (28), we also examined the relationship between task-related activity in MT+ and retardation score. To further investigate task-related activation patterns in MDD patients, we incorporated psychophysiological interactions (PPI) analysis to explore task-modulated FC based on MT+.

## 2 Materials and methods

### 2.1 Participants

We initially recruited 24 MDD patients and 25 healthy adults who were matched for age and gender. The MDD participants were recruited from Hangzhou Seventh People’s Hospital. All the subjects participated in MRI and psychophysical experiments. All individuals had an education background above the college degree, and normal or corrected-to-normal vision. Criteria for MDD inclusion were: (i) presence of an acute depressive episode and the diagnosis MDD in accordance with the Diagnostic and Statistical Manual of Mental Disorders, Fifth Edition (DSM-V) as (a) established by the assessing psychiatrist, and (b) confirmed with Mini International Neuropsychiatric Interview (M.I.N.I.) (29) (ii) clinical symptoms of depression as measured by a Hamilton Depression Rating Scale (HAMD-17) ≥ 17; (iii) receiving treatment with selective serotonin reuptake inhibitors (SSRIs). Exclusion criteria included: (i) any other psychiatric disorder, or a mental disorder caused by a physical illness or substance abuse or a personality disorder; (ii) history of traumatic brain injury, epilepsy, or other known organic lesion of the central nervous system; (iii) psychotic features in depressive episodes; and (iv) history of endocrine disease or blood, heart, liver, kidney dysfunction, another medical disorder such as diabetes, or pregnancy. The study received approval from the Ethics committee of Hangzhou Seventh People’s Hospital. Written informed consent was obtained from all the participants.

To reduce the effects of head motion on task-state fMRI results, subjects with translations greater than 1.5 mm or rotations greater than 1.5° in each direction were excluded: exclusion of 4 HC subjects and 4 MDD subjects. After these exclusions, 20 MDD patients and 21 HC subjects remained in our present study sample (**Table 1**). Among them, 17 MDD patients and 15 HC subjects completed the psychophysical experiment.

**Table 1:** Demographic information of participants and clinical data of patients.

Variables	Healthy controls (*n* = 21)	MDD patients (*n* = 20)	*P* value
Gender (M/F)	6/15	4/16	0.523^a^
Age, years (SD)	23.8 (2.4)	25.0 (5.3)	0.345^b^
HAMD-17 score (SD)	–	19.3 (3.1)	–
Retardation score (SD)	–	6.3 (1.1)	–
Treatment, n (%)			
Antidepressants		15 (75.0)	
Antipsychotics		6 (30.0)	
Mood stabilizers		3 (15.0)	
Benzodiazepines		7 (35.0)	

HAMD, Hamilton Depression Rating Scale; MDD, major depressive disorder; SD, standard deviation.

a Chi-square test.

b Two-sample t-test.

Retardation score was calculated by summing the scores of subitems 1,7,8,14 of the HAMD-17 scale.

### 2.2 Measurement of visual motion perception

All stimuli were created using Psychophysics Toolbox (30) based on MATLAB (MathWorks, Natick, MA, USA) and displayed on a linearized monitor (1920 × 1080 resolution, 100-Hz refresh rate, Cambridge Research System, UK). Participants viewed the stimuli from a distance of 72 cm, with their heads stabilized by a chinrest. Stimuli appeared on a gray (56 cd/m^2^) background.

The details of the procedure for measurement are available in our recent studies (7, 28). Briefly, stimuli with diameter of 2° and 10° were vertically drifting sinusoidal gratings with high contrast (contrast: 50%; spatial frequency, 1 cycle/°; speed, 4°/s) (see **Supplementary Figure S1** in 7). The edge of the grating was blurred with a raised cosine function (width, 0.3°). The grating was ramped on and off with a Gaussian temporal envelope, and the grating duration was defined as 1 standard deviation (SD) of the Gaussian function. The duration was adaptively adjusted in each trial on a staircase procedure (three-down/one-up staircases) to estimate the duration thresholds. Thresholds for large and small gratings were obtained from a 160-trial block that contained four interleaved. Stimulus demonstration and practice trials were presented before the first run. Auditory feedback was provided for each wrong response. The psychophysical experiment included 80 trials each of 2° and 10° stimuli, and recorded the grating duration per trial. For each participant, the correct rate for different stimulus durations was computed for 2° stimuli. These values were then fitted to a cumulative Gaussian function, and the duration threshold corresponding to the 75% correct point on the psychometric function was estimated for 2° stimulus size. The analysis in this study does not involve the 10° stimuli, as the perception of these stimuli by subjects is influenced by spatial suppression (4, 5, 7).

### 2.3 MR acquisition

**Figure 1:**
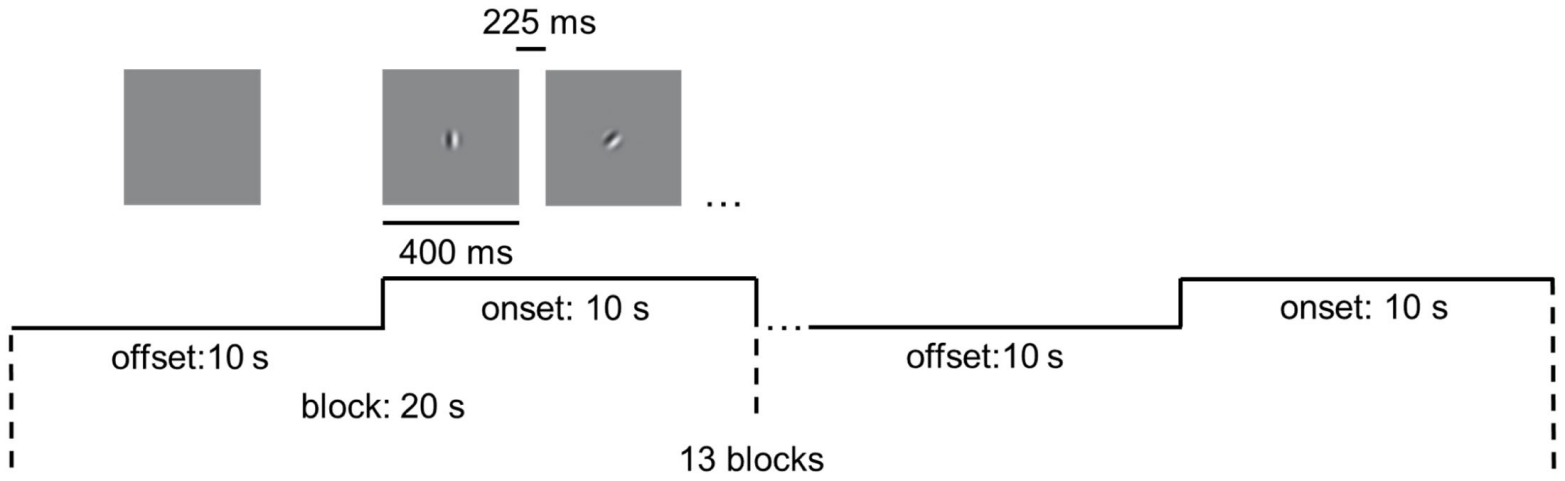
Experimental design of the visual motion perception task. The task included 13 blocks. Each block contained 10 s blank (offset) and 10 s stimulus presentation (onset). Drifting gratings (400 ms on, 225 ms blank) were presented within onset.

We performed magnetic resonance imaging (MRI) experiments in a 7T whole body MR system (Siemens Healthcare, Erlangen, Germany) with a Nova Medical 32 channel array head coil. Sessions included block-design task-state fMRI and structural image scanning. Ear plugs and foam pads were used to minimize noise and head motion when scanning. Task-state scans were acquired with 1.5-mm isotropic resolution (transverse orientation, TR/TE = 2000/20.6 ms, 130 volumes, slice number = 110, flip angle = 70°). As shown in **Figure 1**, in the task-state fMRI, thirteen blocks were presented during a run (20 s each, 130 TRs total). Each block contained 10 s baseline (offset) and 10 s stimulus presentation (onset), the latter comprising 16 stimuli of 400 ms each, interspersed with 16 intervals of 225 ms. The stimulus is a moving grating (contrast = 98%, speed = 2°/s, diameter = 2°, spatial frequency =3 cycle/°), moving in one of eight possible directions in a randomized and count-balanced order. Furthermore, within the task, participants were required to respond by pressing a key when red dots appeared randomly at the center of the screen, aiming to enhance their focus. Visual stimuli were presented using the functional magnetic resonance experiment system (SMARTEC, SA-9800) from Shenzhen Virtue Medical. Participants with myopia were instructed to wear magnetic resonance-compatible glasses to guarantee a corrected visual acuity greater than 1.0. Structural images were acquired using a MP2RAGE sequence (TR/TI1/TI2 = 5000/901/3200ms) with 0.7-mm isotropic resolution.

### 2.4 Task-state fMRI data processing and analysis

Task-state fMRI data were preprocessed using Data Processing & Analysis for Brain Imaging (DPABI) Version 6.2_220915 (31) on MATLAB R2020b. During the preprocessing stage, the images were first realigned. To reduce the impact of head movements on the result of task-state fMRI, participants were excluded when movement parameters exceeded 1.5 mm or 1.5° in any direction. After the step, 4 patients and 4 healthy controls were excluded. Then the fMRI images were co-registered with the high-resolution anatomical images, normalized to MNI (Montreal Neuroimaging Institute) space using Diffeomorphic Anatomical Registration through Exponentiated Lie algebra algorithm (DARTEL) with a resolution of 1.5 mm^3^, and spatially smoothed with 3 mm full-wide-half-maximum (FWHM) Gaussian kernel.

Regions of interests (ROIs) were defined for each hemisphere in 2 anatomical regions: MT+ defined by a cytoarchitectonically probabilistic map (32), and V1 defined by Brodmann Area 17 (BA17) mask (**Supplementary Figure S** **1**). The inclusion of the V1 as a control region further supports the regional specificity of the results. The calculation of percent signal change was consistent with the method of previous studies (5, 33). Before calculation of percent signal change, average time courses were obtained from each ROI utilizing DPABI in MATLAB. The data were segmented into epochs starting from the beginning of each block to 2 seconds after the start of the next block, totaling 22 s. The baseline response was defined as the average signal from 6-10 s of all epochs. Subsequently, the time course data was converted into a percent signal change series by dividing the signal at each time point by the baseline signal and then multiplying by 100. For each subject, the percent signal change of each ROI was defined as the average signal change from 18-22 s of all epochs.

Voxel-based first-level analysis of task-state fMRI was conducted using the General Linear Model (GLM). A design matrix was established for each participant, reflecting the two states (baseline and visual stimulus presentation) in the visual motion perception task. The blocks were convolved with a canonical hemodynamic response function. To account for head movements, the head motion parameters of six directions were included as regressors in the GLM. Subsequently, the regression coefficients (beta values) were estimated for each voxel. The first beta value was considered relevant to the visual task. Then, for each subject, the mean first beta value of all voxels within the ROI was taken as the beta value of this ROI.

### 2.5 Functional connectivity analysis

The dynamic functional connectivity (FC) with MT+ as the seed region was calculated using the PPI analysis (34) based on SPM12 (https://www.fil.ion.ucl.ac.uk/spm/software/spm12/). PPI analysis was based on the SPM.mat file generated from the previous GLM step, which contains the task design information, including respective onset time series and duration for condition 1 (onset: stimulus presentation) and condition 2 (offset: baseline). Next, the time series of seed ROI (left and right MT+) were extracted. A PPI model was created for each subject, comprising three main components: the physiological component which corresponded to the time series of the seed region left and right MT+, the psychological component which corresponded to the conditions of task (onset and offset), and the PPI component which reflected the interaction between the psychological and physiological variables. The association between the psychological component and physiological component was achieved by constructing an interaction term. The PPI calculation involved element-by-element multiplying the deconvolved time series of the seed region by a vector representing task conditions (35, 36). PPI was derived for each subject by contrasting the onset and offset. Beta weights map of the PPI component was generated for each subject used in two-sample *t*-test across the entire brain.

### 2.6 Statistical analysis

In the study, outliers were defined as values that exceed 1.5 times the interquartile range and removed before statistical analysis. One sample *t*-test was conducted to investigate the task-related activation in the whole brain within MDD or HC group using DPABI. Other statistical analysis was performed using SPSS 26 (IBM, USA). Student’s *t* test was used to examine the differences between MDD group and HC group. Pearson’s correlation coefficients were calculated to analyze the relationship between imaging indicators, behavior measures and symptoms of patients. Differences or correlations were considered statistically significant if *P* < 0.05. Corrections for multiple comparisons were conducted using false discovery rate (FDR) correction. All analyses were adjusted for medication effects (see Limitation for more details).

## 3 Results

### 3.1 Demographic and clinical data

As shown in **Table 1**, after quality control of the head movement, 20 MDD patients and 21 HC subjects remained in our present study sample. According to results of chi-square test for gender and two-samples *t*-test for age, there were no significant difference in gender (*Z* = 0.963, *P* = 0.336) and age (*T* = 1.055, *P* = 0.298) between MDD and HC groups.

### 3.2 Abnormal visual motion perception in MDD

**Figure 2:**
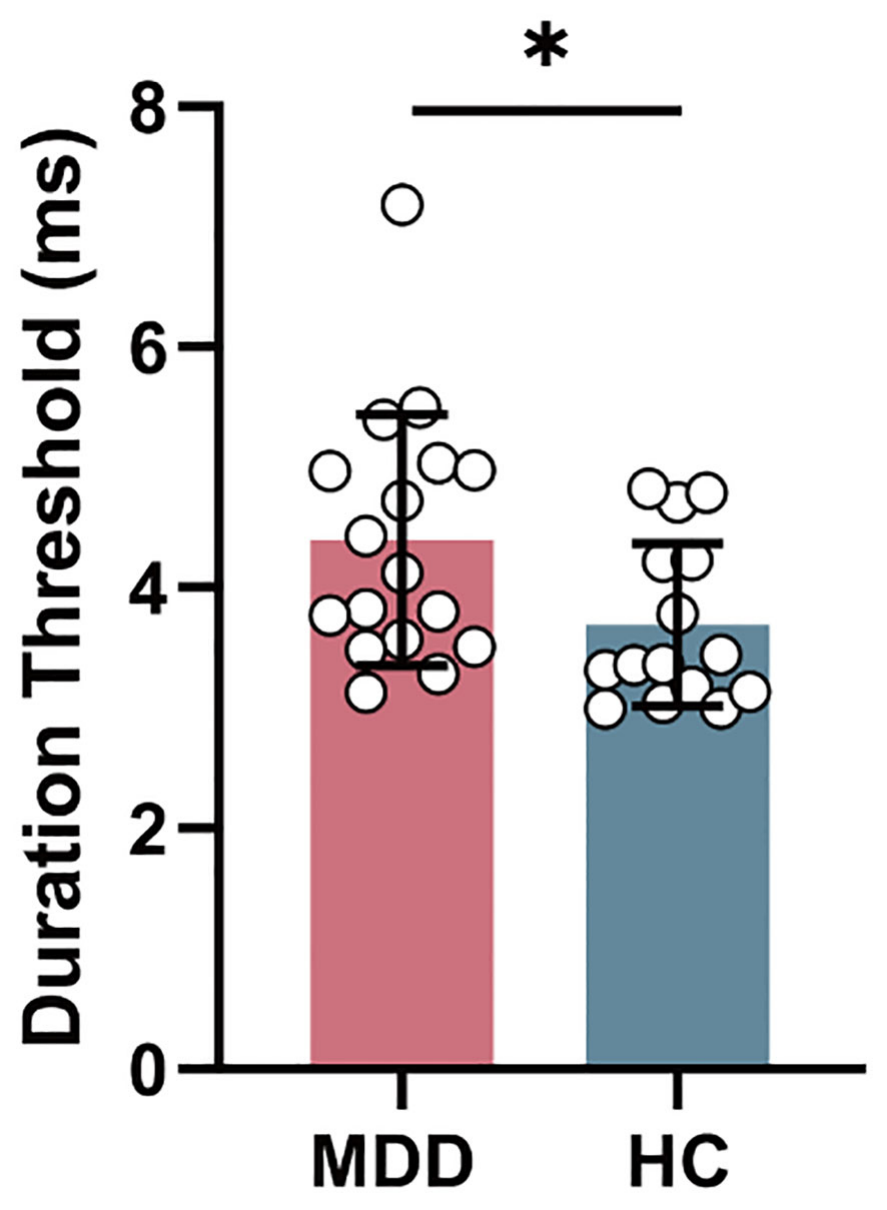
The intergroup difference in duration threshold between MDD and HC groups. MDD, major depressive disorder; HC, healthy control. *P < 0.05.

In behavior level, duration threshold in MDD group significantly increased (*T* = 2.216, *P_FDR_* = 0.034) compared to HC group (**Figure 2**).

### 3.3 Reduced activity in MT+ of MDD

**Figure 3:**
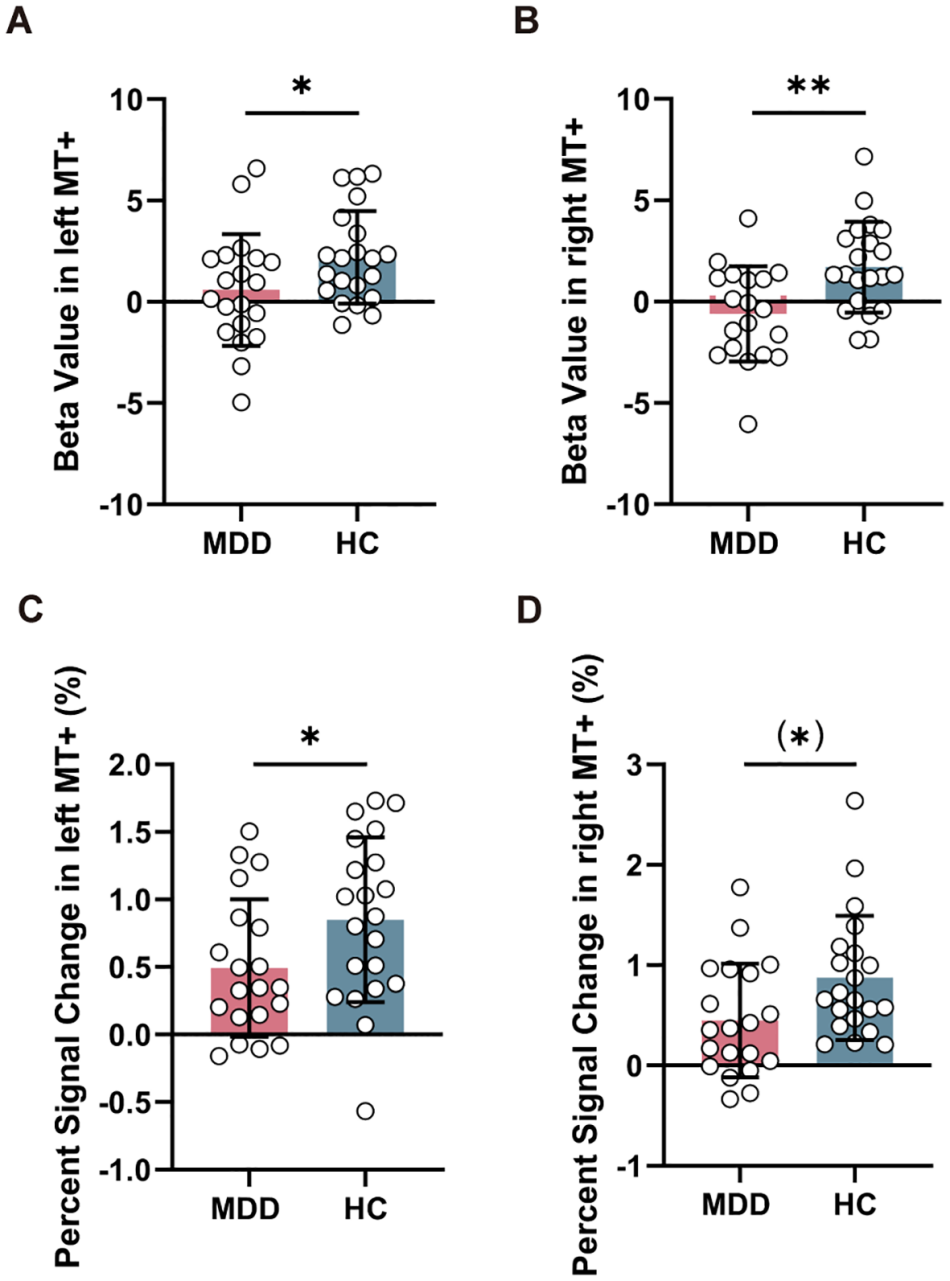
Comparison of task-related activation in MT+ between MDD and HC groups. Reduced beta value **(A, B)** and percent signal change **(C, D)** in left and right MT+ in MDD compared to HC group. MT+, middle temporal complex; MDD, major depressive disorder; HC, healthy control. *P_FDR_ < 0.05, **P_FDR_ < 0.01.

Compared to HC group, MDD exhibited reduced beta value in left MT+ (*T* = -2.026, *P_FDR_* = 0.049) (**Figure 3A**) and right MT+ (*T* = -3.188, *P_FDR_* = 0.006) (**Figure 3B**). However, there was no significant change in beta value within left V1 (*T* = -0.258, *P_FDR_* = 0.798) and right V1 (*T* = -1.808, *P_FDR_* = 0.157) between MDD and HC groups (**Supplementary Figure S2**). Furthermore, MDD group showed decreased percent signal change in left MT+ (*T* = -2.035, *P_FDR_* = 0.049) (**Figure 3C**) and nearly significant in right MT+ (*T* = -2.294, *P_FDR_* = 0.054) (**Figure 3D**), but no significant change in left V1 (*T* = 0.377, *P_FDR_* = 0.708) and right V1 (*T* = 0.052, *P_FDR_* = 0.959) (**Supplementary Figure S2**).

### 3.4 Relationship of activity in MT+ and visual motion perception

**Figure 4:**
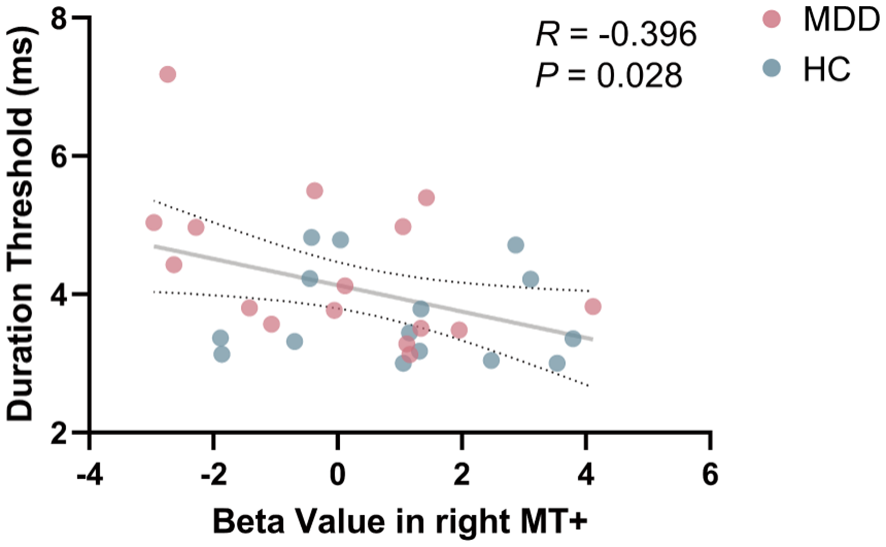
Correlation between beta value in right MT+ and duration threshold in the entire cohort including MDD and HC. MT+, middle temporal complex; MDD, major depressive disorder; HC, healthy control.

In order to explore whether the phenomenon of MT+ activation intensity related to visual perception in HC group (33) could be extended to the MDD group, Pearson’s correlation analysis was conducted. When combining the data from both MDD and HC groups for correlation analysis, a significant negative correlation between beta value in right MT+ and duration threshold was found (*R* = -0.396, *P* = 0.028) (**Figure 4**). No significant correlation between beta value in left MT+ and duration threshold (*R* = -0.028, *P* = 0.879) (**Supplementary Figure S3**). In V1, beta value and percent signal change were not significantly related to duration threshold (*P* > 0.05) (**Supplementary Figure S4**).

### 3.5 Activity in MT+ relates to psychomotor retardation in MDD

**Figure 5:**
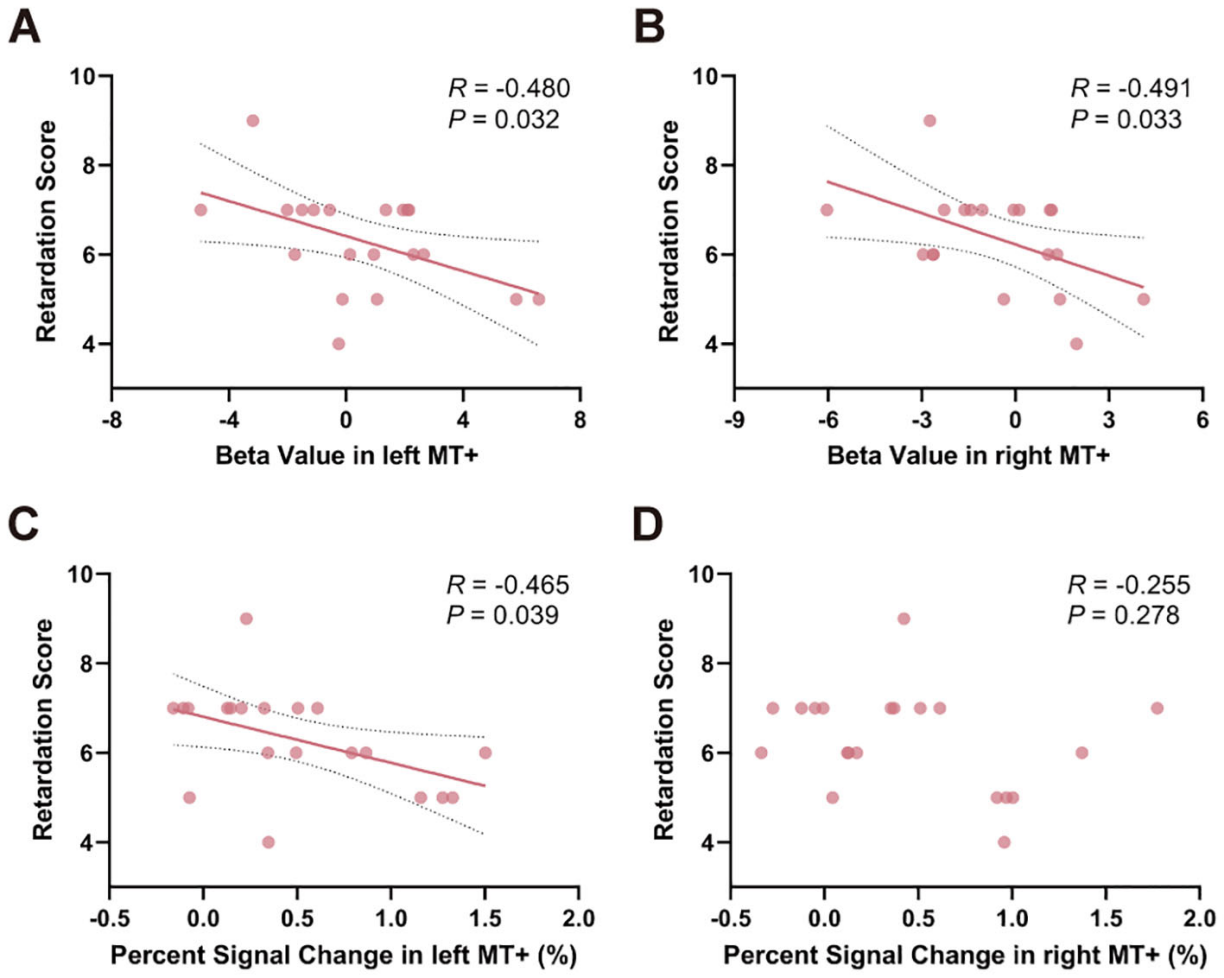
Relationships between task-related activation in MT+ and psychomotor retardation score within MDD group. Significant correlation between beta value in both left **(A)** and right MT+ **(B)** and psychomotor retardation score. **(C)** Percent signal change in left MT+ significantly correlated with psychomotor retardation score. **(D)** Percent signal change in right MT+ and psychomotor retardation score were not significantly correlated. MT+, middle temporal complex; MDD, major depressive disorder.

To examine the relationship between task-related activity and psychomotor retardation, Pearson’s correlation analysis was used to test the correlation between activity, retardation score and HAMD-17 total score. Relationships between beta value in left MT+ (*R* = -0.480, *P* = 0.032) and right MT+ (*R* = -0.491, *P* = 0.033) and retardation score were significantly (**Figure 5A, B**). Percent signal change in left MT+ and retardation score was significantly negatively correlated (*R* = -0.465, *P* = 0.039) (**Figure 5C**). Percent signal change in right MT+ did not relate to retardation score (*R* = -0.255, *P* = 0.278) (**Figure 5D**). As a control region, there was no significant correlation between V1 and the retardation score, either in terms of beta value or percent signal change (**Supplementary Figure S5**). Moreover, no significant results were found when correlating the activity of MT+ and V1 with HAMD-17 total score (*P* > 0.05) (**Supplementary Figure S6**).

### 3.6 Altered functional connectivity with MT+ as the seed region in MDD

**Figure 6:**
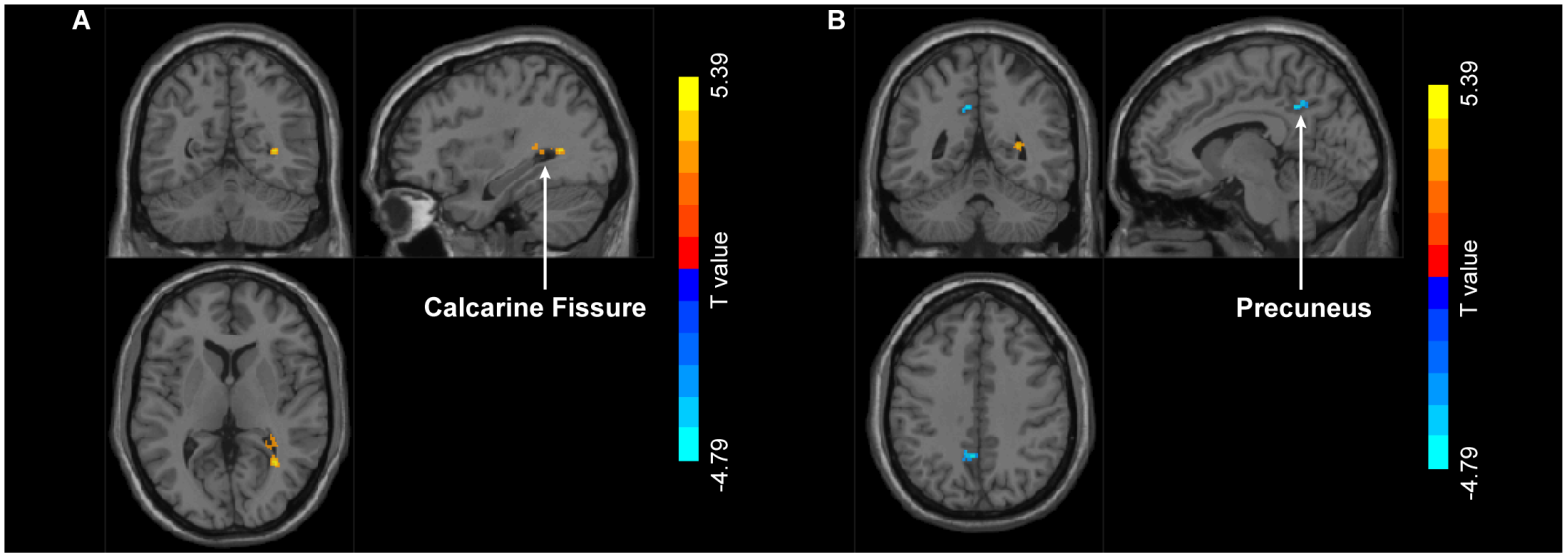
Group differences in FC with left MT+ as the seed region between MDD and HC. Compared to HC group, MDD exhibit increased FC in right calcarine fissure and surrounding cortex **(A)** and decreased FC in left precuneus **(B)**. MT+, middle temporal complex; MDD, major depressive disorder; HC, healthy control.

PPI analysis revealed that when left MT+ was set as the seed region, significant intergroup differences emerged in right calcarine fissure and surrounding cortex and left precuneus (**Figure 6**). Specifically, compared to HC group, MDD patients showed significantly increased FC between left MT+ and right calcarine fissure and surrounding cortex, and decreased FC between the left MT+ and the left precuneus (**Table 2**). These FC were not significantly related to behavioral duration threshold both in MDD and HC groups (*P* > 0.05) (**Supplementary Figure S7**). However, when the seed region was set to right MT+, no significant intergroup differences were observed.

**Table 2:** Significant altered brain regions in FC between MDD and HC groups using left MT+ as the seed region.

Seed		Region	BA	Cluster size	Peak *t*-value	MNI coordinates		
						X	Y	Z
left MT+	MDD > HC	right calcarine fissure and surrounding cortex	\	157	5.4	33	-57	6
	MDD < HC	left precuneus	BA7	116	-4.8	-7.5	-48	37.5

FC, functional connectivity; MT+, middle temporal complex; MDD, major depressive disorder; HC, healthy controls; BA, Brodmann Area; MNI, Montreal Neurological Institute.

## 4 Discussion

This multi-modal study is the first to investigate the neural activation pattern of MT+ and V1 in MDD patients during grating motion perception task. Compared to HC participants, duration threshold in psychophysical experiment was increased and activity in MT+ in task-state fMRI were reduced in MDD group. In the combined MDD and HC sample, a significant negative correlation was found between beta value in right MT+ and duration threshold. However, no activation differences were noted in the V1. Additionally, in MDD group, MT+ activity significantly correlated with the retardation score. With left MT+ as the seed, the MDD group exhibited greater FC with the right calcarine fissure and surrounding cortex and reduced FC with the left precuneus compared to HC group. This study reveals differences in behavior and brain function of MDD patients when processing visual motion information. The results fill the gap left by the absence of task-related modalities in previous research, and a link was discovered between behavioral performance and task-related activity in MDD patients. The significance of these findings lies in their potential to enhance the understanding of the neural mechanisms underlying altered motion perception in depression, particularly how specific brain regions like MT+ and their connectivity are impacted in MDD.

Reduced task-related activity in MT+ of MDD patients may indicate specific neurobiological changes. Our previous ultra-high field magnetic resonance spectroscopy (MRS) study found that MDD group had reduced concentrations of various neuro-metabolites including Glu and GABA, and identified abnormal excitation-inhibition balance with decoupling of inhibitory GABA from excitatory glutamate (7). Studies on the whole-brain structure of MDD patients have found reductions in gray matter volume (37–40) and cortical thickness (41, 42) within visual cortex. A multi-site fMRI study discovered that the topological structure of functional brain networks, including the visual network, was disrupted in MDD, with decreased degree of nodes (43). The abnormal brain activity in MT+ during task may result from an interaction of neurotransmitter imbalance, brain structural changes, and abnormal connectivity in functional networks, forming multi-level brain alterations. This provides new insights into the biological basis of MDD.

Consistent with previous findings (7, 44), this study discovered that visual motion perception is impaired in MDD patients, and within a sample group including both MDD and HC, the performance of psychophysical visual perception task is significantly correlated with neural activity in task-state fMRI. MT+ plays a crucial role in processing visual motion information, responsible for identifying and analyzing the motion direction, speed, and trajectory of objects (45, 46). Decreased percent signal change and beta value in MT+ in our result indirectly reflects a reduction in neural activity in this region in MDD patients, potentially impairing their ability to process these motion information (47, 48), thereby affecting the accuracy and efficiency of motion perception. Our finding on the relationship between neural activity in MT+ and visual motion perception corroborate previous study in HC group (33) and further extend to the MDD cohort. This suggests that although visual motion perception functions and neural activity in MT+ are impaired in MDD patients, the correlation between them still exists, MT+ may still be a major contributor to the perception of briefly presented moving stimuli (33). Furthermore, the reduction in MT+ activity may impact emotional regulation and perceptual experience. Changes in MT+ activation may reflect difficulties in processing emotion-related dynamic visual information in MDD patients (49–51), possibly related to negative emotional experiences (52, 53), social difficulties (54), an exaggerated response to negative stimuli (55, 56), or an insufficient response to positive stimuli (57). Visual motion perception involves the brain’s processing of dynamic information, not limited to the visual system but also including integration with other sensory systems (58). Potential impairments in MT+ for MDD patients may have widespread effects on daily functioning and emotional states.

Our previous resting-state fMRI study (28) found that MDD patients exhibited abnormally increased ALFF in the left MT+. While in this study, the activity of MT+ in MDD group decreased during perception task. And both findings were significantly correlated with retardation score. The changes in activation of MT+ in both resting and task states reveal the brain’s differential processing of dynamic information under various states in MDD patients (59). In the resting state, the brain is not in a complete rest but is engaged in a series of internal processing activities, such as memory consolidation (60) and emotion regulation (61). The increased ALFF in left MT+ during the resting state in MDD patients may reflect their brain’s overactivity in the absence of specific external stimuli (62), which could be related to the patients’ persistent negative thinking and rumination (63–65). During task performance, the brain typically increases activity in relevant areas to process the task (59). However, the decreased activity of MT+ in MDD patients during task may indicate a reduced efficiency in processing external dynamic visual information (66, 67). This might be due to the inability of individuals with depression to mobilize brain resources as effectively as healthy individuals during task execution (68).

Psychomotor retardation is one of the common symptoms of MDD, involving slowness in thought processing, speech, and physical movements (3, 69, 70). In this study, compared to HC group, beta value and percent signal change in both left and right MT+ were decreased in MDD patients in the between-group comparison. It indicates that task-related activity of both left and right MT+ are impaired in MDD patients. Additionally, the impaired activity was significantly correlated with psychomotor retardation score, suggesting a specific link between brain activity patterns and the symptoms of psychomotor retardation in depression (12, 28, 71).

PPI analysis aims to explore whether FC between brain regions change under specific task conditions (34), measuring task-dependent dynamic FC. In this analysis, we conducted statistical analyses across the whole brain based on the seed region left MT+, and identified calcarine fissure and surrounding cortex and precuneus as key regions showing abnormal changes in MDD patients. Firstly, the calcarine fissure and surrounding cortex is involved in the primary processing of visual information (72, 73). MDD patients exhibit various structural abnormalities in this region, such as cortical thinning (74), reduced gray matter volume (75), and abnormal nodal efficiency (76, 77). We observed increased FC between left MT+ and right calcarine fissure and surrounding cortex, suggesting that MDD patients may require more neural resources when processing visual motion information (78), or may exhibit abnormal neural activity patterns. This enhanced connectivity may serve as a compensatory mechanism for perceptual processing impairments (79). Additionally, FC between left MT+ and right precuneus weakened, particularly in the Brodmann Area 7 (BA7). BA7, located in the parietal lobe, is crucial for processing visual inputs, involving the integration of bodily sensations and visual information, as well as regulating attention, visual-motor coordination, and self-awareness functions (80, 81). Previous studies found reduced cortical surface area of precuneus in MDD patients (82), accompanied by abnormal functional connectivity with multiple brain regions (83–86). The weakened FC between MT+ and precuneus may affect the effective processing and integration of task-related visual motion information and self-relevant information. It is worth emphasizing that the calcarine fissure and surrounding cortex, MT+, and the precuneus all play significant roles in visual processing. Therefore, abnormalities in network function from visual perception to visual information integration may impair MDD patients’ ability to process visual stimuli. This underscores the importance of in-depth research into MDD from visual cortex to better understand the neural mechanisms of depression. But in PPI analysis, we found that only dynamic FC of left MT+ showed between-group differences, while right MT+ did not. This possibly reflects an asymmetry in task-related connectivity in MT+.

Observing abnormalities in the activity of MT+ through imaging techniques may offer new perspectives for the diagnosis and treatment of MDD. Compared to other psychiatric disorders, the specific activation patterns within this region in MDD patients could serve as biomarkers for auxiliary diagnosis (87). Understanding the abnormal activations in MT+ of the brains of MDD patients can aid in developing new therapeutic methods. For instance, targeting the neural circuits that affect the function of MT+ through methods such as neurofeedback (88, 89) and physical stimulation (25) might provide more personalized and precise treatment options for MDD.

This study builds upon previous multi-modal research on depression in MT+ by further measuring the abnormalities in beta value, percent signal change and FC in MT+ of MDD patients during visual motion perception task. It found that task-related activation decreased and was significantly correlated with psychophysical performance and psychomotor retardation. And MDD patients exhibit abnormalities in the internal connectivity within the visual functional network. In summary, our results not only enhance the understanding of the neural mechanisms behind visual motion perception impairments in MDD, but also further support MT+ as a candidate biomarker region for MDD.

## 5 Limitations and future directions

We admit that the main limitation of the present study is the potential confounding influence of pharmacological treatments. Indeed, the majority of the acute MDD patients in our cohort were taking medications, including mood stabilizers, antipsychotics, antidepressants, and benzodiazepines, which may possibly affect results. Following recent suggestions and standards, we examined the potential impact of the psychotropic medication load—the number and dosage of different medications, we then used the codes 0, 1, 2, and 3 to indicate no medication, and dose-equivalents below, equal, or above the average effective daily dose, respectively (90). A composite measure of the medication load was calculated by summing all individual medication codes across each category and for each MDD patient. We explored the possible influence of medications on behavior and fMRI data by correlating the resulting medication load with duration threshold, beta value and percent signal change in left and right MT+. The medication load did not correlate with this measure in antidepressants, antipsychotics, mood stabilizers, and benzodiazepines (*P* > 0.05).

Subsequently, to further control for an eventual effect of pharmacotherapy on duration threshold, beta value and percent signal change within left and right MT+, we conducted two-sample *t*-test to compare the variables for each medication class (mood stabilizers, antidepressants, benzodiazepines, and antipsychotics), between those patients who were in treatment with the respective drug and those who were not. We found no differences between patients who were in treatment with mood stabilizers (*n* = 3) and patients who were not (*n* = 17) (*P* > 0.05), between patients who were in treatment with antidepressants (*n* = 15) and patients who were not (*n* = 5) (*P* > 0.05), between patients who were in treatment with benzodiazepines (*n* = 7) and patients who were not (*n* = 13) (*P* > 0.05), as well as between patients who were in treatment with antipsychotics (*n* = 6) and patients who were not (*n* =14) (*P* > 0.05).

Yet another limiting factor is the rather low subject number. The limited number of subjects may reduce the statistical power of the study, especially for correlation analysis. Furthermore, the cross-sectional design employed in our study does not allow for tracking changes in patients across these imaging indicators in MT+. Nevertheless, although our research has combined psychophysical experiment with task-state fMRI measurements, it still lacks data from modalities such as structural neuroimaging and resting-state fMRI, which limits the scope of the study. Integrating multi-modal types of data, such as resting-state fMRI, structural neuroimaging, molecular, and physiological measurements, can provide convergent evidence from different domains, thereby enhancing the robustness and depth of conclusions. In sum, while the current study contributes valuable insights into MT+ as potential biomarker target of MDD, its limitations highlight the necessity for further research that employs larger sample sizes, longitudinal designs, and more multi-modal approaches.

## Data Availability

The original contributions presented in the study are included in the article/**Supplementary Material**. Further inquiries can be directed to the corresponding authors.
