# Supplementary material for: Abnormal activation patterns in MT+ during visual motion perception in major depressive disorder
Source: Front Psychiatry. 2024 Aug 26;15:1433239. doi: 10.3389/fpsyt.2024.1433239 (PMC11381256; doi:10.3389/fpsyt.2024.1433239)
Supplement: Supplementary file 1 [file DataSheet1.docx]

Supplementary Material

# Supplementary Figures


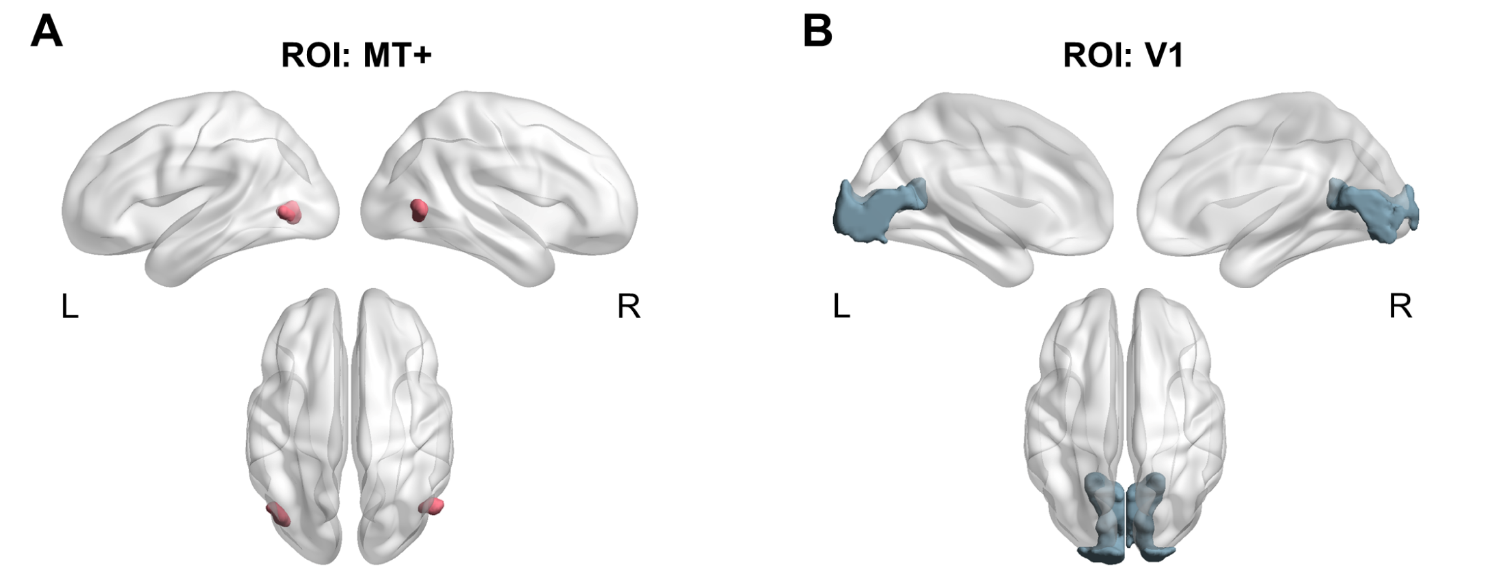


**Figure S1.** The templates used for task-related fMRI data. Masks of ROIs: MT+ (A) and V1 (B). ROI, regions of interests; MT+, middle temporal complex; V1, primary visual cortex.


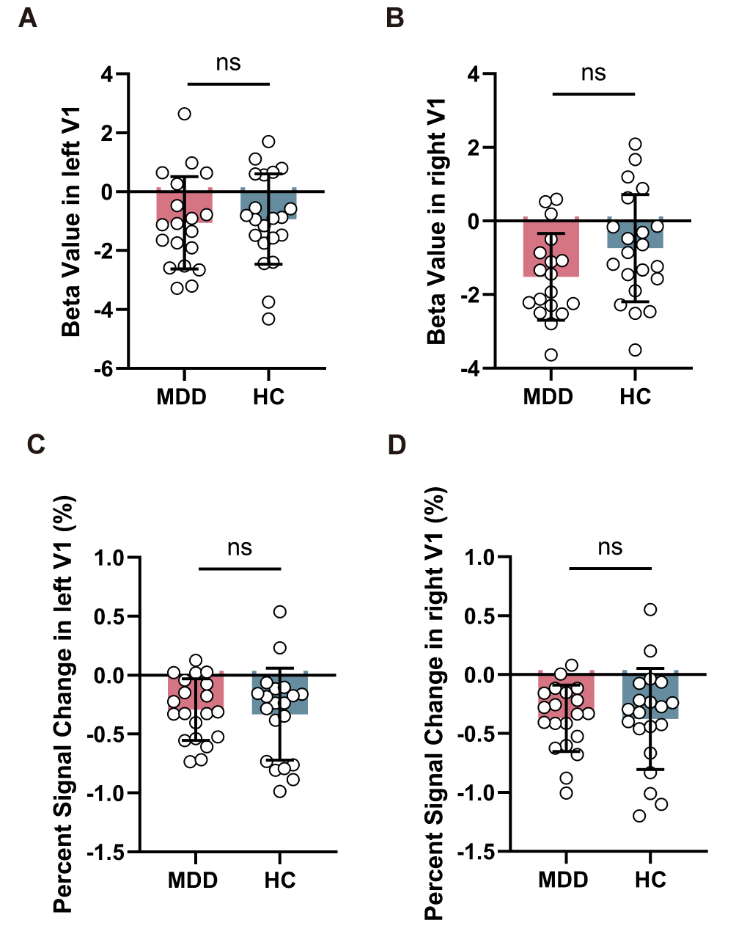


**Figure S2.** Comparison of activation in V1 between MDD and HC groups. No significant difference in beta value (A-B) and percent signal change (C-D) in left and right V1 between MDD and HC groups. V1, primary visual cortex; MDD, major depressive disorder; HC, healthy control.


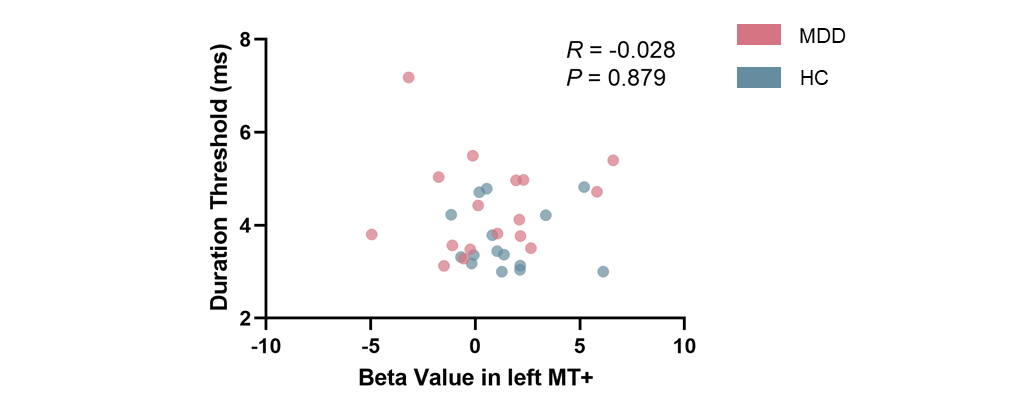


**Figure S3.** Correlation between beta value in left MT+ and duration threshold. MT+, middle temporal complex; MDD, major depressive disorder; HC, healthy control.


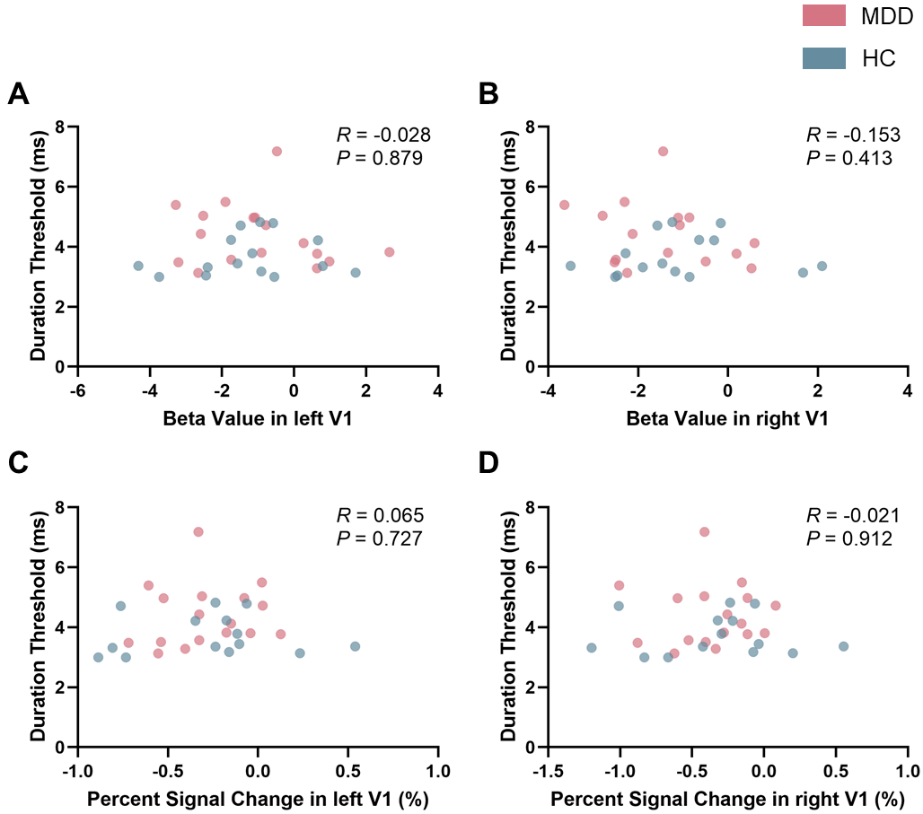


**Figure S4.** Correlations between activity in left and right V1 and duration threshold. No significant correlation between beta value (A-B) and percent signal change (C-D) in V1 and duration threshold. V1, primary visual cortex; MDD, major depressive disorder; HC, healthy control.


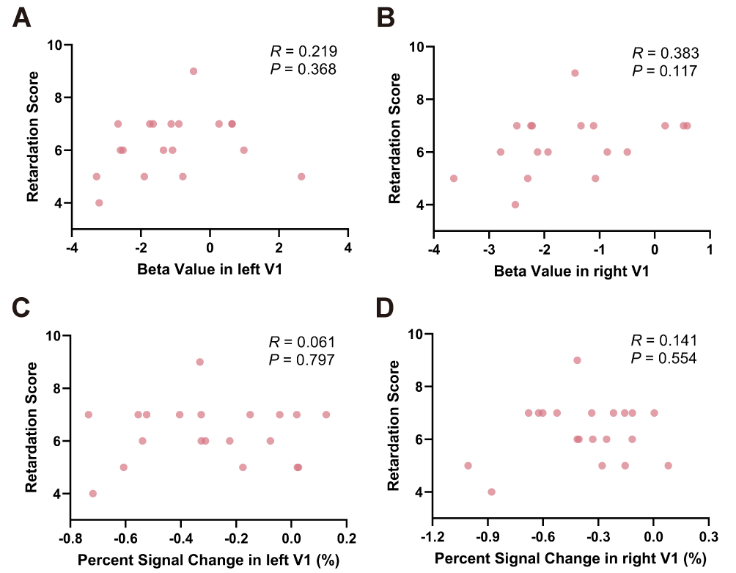


**Figure ~~S3~~S5.** Relationships between task-related activation within V1 and psychomotor retardation score within MDD group. No significant correlation between beta value in left (C) and right V1 (D) and psychomotor retardation score. Percent signal change in left V1 (A) and right V1 (B) were also not related to psychomotor retardation score. V1, primary visual cortex; MDD, major depressive disorder.


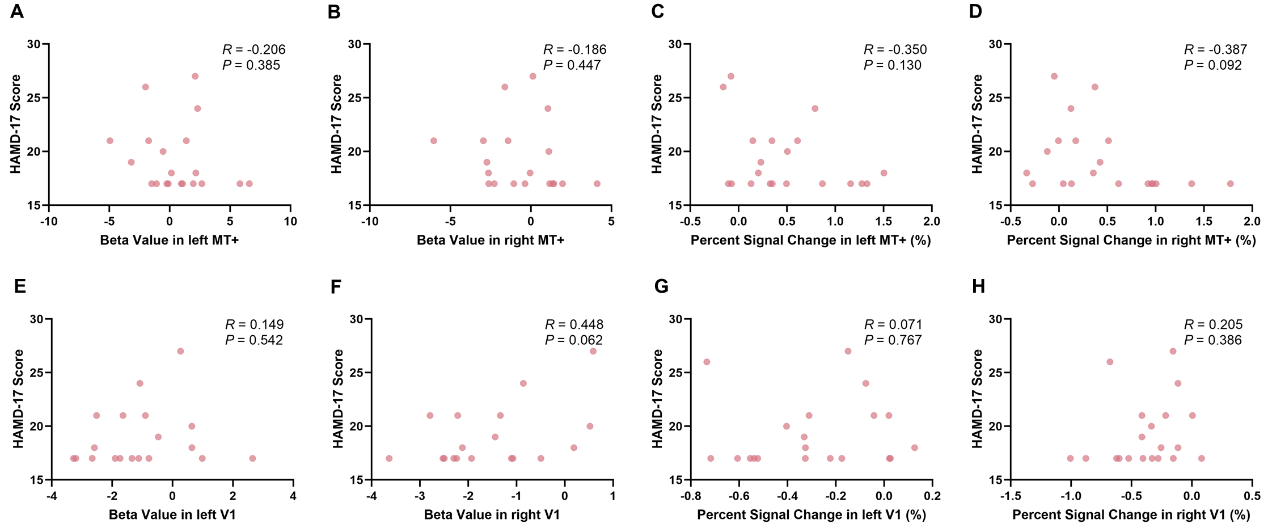


**Figure S6.** Correlations between activity in MT+ and V1 and HAMD-17 score in MDD group. No significant correlation in MT+ (A-D) and V1 (E-H). MT+, middle temporal complex; V1, primary visual cortex; MDD, major depressive disorder.


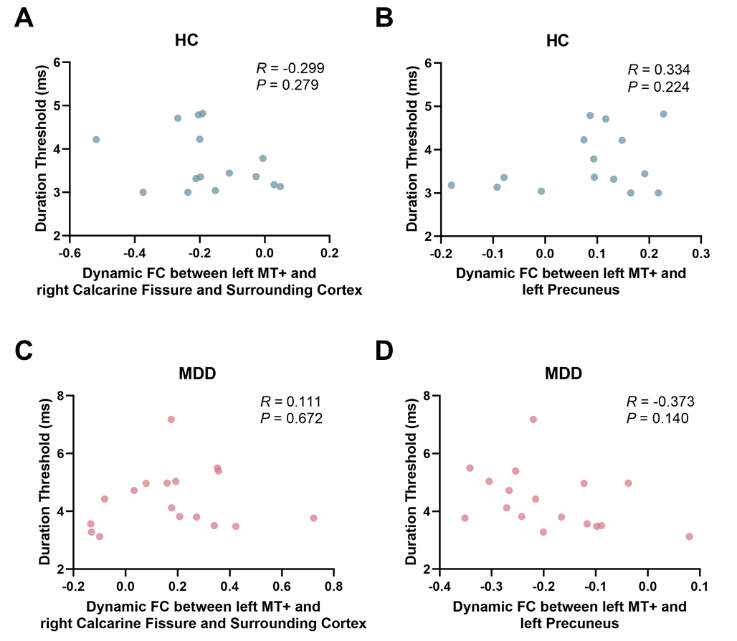


**Figure S7.** Relationships between FC and duration threshold. No significant correlation between FC and duration threshold in HC (A-B) and MDD groups (C-D). FC, functional connectivity; MT+, middle temporal complex; MDD, major depressive disorder; HC, healthy control.
